# Revisiting the J shaped curve, exploring the association between cardiovascular risk factors and concurrent depressive symptoms in patients with cardiometabolic disease: Findings from a large cross-sectional study

**DOI:** 10.1186/1471-2261-14-139

**Published:** 2014-10-28

**Authors:** Bhautesh Dinesh Jani, Jonathan Cavanagh, Sarah JE Barry, Geoff Der, Naveed Sattar, Frances S Mair

**Affiliations:** CSO Clinical Fellow, General Practice and Primary Care, Institute of Health and Wellbeing, College of Medical, Veterinary and Life Sciences, University of Glasgow, Glasgow, G12 9LX UK; Professor of Psychiatry, Mental Health and Wellbeing, Sackler Institute, Institute of Health and Wellbeing, College of Medical, Veterinary and Life Sciences, University of Glasgow, Neurology Block, Southern General Hospital, Glasgow, G51 4TF UK; Consultant Biostatistician, Robertson Centre for Biostatistics, Institute of Health and Well Being, College of Medical, Veterinary and Life Sciences, University of Glasgow, Glasgow, G12 8QQ UK; Statistician, MRC/CSO Social and Public Health Sciences Unit, Institute of Health and Wellbeing, College of Medical, Veterinary and Life Sciences, University of Glasgow, Glasgow, G12 8RZ UK; Professor of Metabolic Medicine, BHF Glasgow Cardiovascular Research Centre, Institute of Cardiovascular and Medical Sciences, College of Medical, Veterinary and Life Sciences, University of Glasgow, Glasgow, G12 8TA UK; Professor of Primary Care Research, General Practice and Primary Care, Institute of Health and Wellbeing, College of Medical, Veterinary and Life Sciences, University of Glasgow, Glasgow, G112 9LX UK

**Keywords:** Cardiovascular risk factors, J-curve, Depression, Blood pressure, Body mass index, Total cholesterol, HbA1C, Diabetes, Stroke, Coronary heart disease

## Abstract

**Background:**

Depression is common in patients with cardiometabolic diseases but little is known about the relationship, if any, between cardiovascular risk factor values and depressive symptoms in patients with these conditions. The objective of this paper is to study the association between cardiovascular risk factors and concurrent depressive symptoms in patients with three common cardiometabolic conditions: coronary heart disease (CHD), stroke and diabetes.

**Methods:**

We retrospectively reviewed primary care data for N = 35537 with 1 of the above 3 conditions who underwent depression screening using the depressive subscale of hospital anxiety and depression score (HADS-D). We reviewed 4 cardiometabolic risk factors (Systolic Blood Pressure [SBP], Diastolic Blood Pressure [DBP], BMI and total cholesterol) recorded concurrently in all patients and HbA1c in patients with diabetes (n = 18453). We analysed the association between individual risk factor value and a positive HADS-D screening result (>7) using logistic regression.

**Results:**

SBP and BMI were noted to have a non-linear “J-shaped” relationship with the probability of having a positive HADS-D and observed nadirs (levels with the lowest probability) of 148 mm Hg and 30.70 kg/m2, respectively. Total cholesterol and DBP found to have a weaker curvilinear association with concurrent depression symptoms and nadirs of 3.60 mmol/l and 74 mmHg. Among patients with Diabetes, HbA1c was also found to have a “J-shaped” relationship with probability of having a positive HADS-D with an observed nadir of 7.06% DCCT. The above relationships remain significant after adjusting for age, sex, socio-economic status and number of co-morbid conditions.

**Conclusion:**

In patients with cardiometabolic disease, cardiovascular risk factor values at both extremes were associated with higher positive depression screening after adjusting for confounders. These findings have potentially important implications for clinical practice in relation to both risk stratification for depression and approaches to secondary prevention in individuals with cardiometabolic disease and merit further investigation to determine the nature and direction of the observed association.

Please see related article: http://www.biomedcentral.com/1741-7015/12/199.

**Electronic supplementary material:**

The online version of this article (doi:10.1186/1471-2261-14-139) contains supplementary material, which is available to authorized users.

## Background

Patients with chronic disease are two to three times more likely to suffer from depression when compared to the general population [1, 2]. It is estimated that depression prevalence is 15-25% in patients with cardio-metabolic diseases such as coronary heart disease (CHD), diabetes and stroke [3–5]. Those with cardiometabolic disease who have suffered from depression have been reported to experience increased adverse clinical outcomes and mortality, and poorer functional abilities [4, 6–8].

In 2008, the American Heart Association Science Advisory recommended routine depression screening for all patients with CHD [9]. However, there is no evidence to date that routine depression screening for patients with cardiometabolic disease leads to any improvement in depression or cardiac outcomes [10, 11]. Moreover, there is some evidence in the UK and US to suggest that routine depression screening for all patients with cardiometabolic disease may struggle to achieve universal coverage [12–14]. In the UK, NICE(National Institute for Health and Care Excellence) recommends that depression screening or ‘case finding’ in patients with chronic disease should be targeted towards those who are believed to be ‘high risk’ [15]; but further research is needed to define who is at ‘high risk’.

The relationship between depression and traditional cardiometabolic disease risk factors such as obesity, hypertension, hyperlipidaemia and raised HbA1c have been studied extensively in the general population. Depression is noted to have a significant positive association with obesity in the general population, with a stronger association noted in females [16, 17]. In addition, evidence from longitudinal studies show that depression may have a bi-directional relationship with obesity [18]. Results from a meta-analysis of prospective cohort studies shows that depression increases the risk of hypertension incidence in the community [19]. A contradictory relationship has been observed between depression and hyperlipidaemia in elderly men and women in the community; with increased prevalence of depressive symptoms observed with low levels of high density lipoprotein cholesterol (higher atherogenic risk) in women and with low levels of low density lipoprotein cholesterol (lower atherogenic risk) in men [20]. In a prospective study of older adults in the general population, the probability of depression increased with raised HbA1c [21]. However, most of the evidence in this area has come from general population studies and there is a paucity of research in those with known cardiometabolic diseases who are likely to be subjected to treatment to reduce these risk factors.

Little is known about the relationship between cardiovascular risk factors and depressive symptoms in those with cardiometabolic disease. The aim of this project is to address this gap by studying the relationship, if any, between a range of cardiovascular risk factors (specifically SBP, DBP, total cholesterol, HbA1c and BMI) and depressive symptoms in those with three cardiometabolic conditions, namely, stroke, diabetes and CHD.

## Methods

### Ethics statement

We received approval from the West of Scotland research ethics committee to undertake this work. The work involved retrospective analysis of a large routinely collected dataset which was completely annonymised and the research team did not have access to patient identifiers, hence individual patient consent was not obtained. NHS Greater Glasgow and Clyde Enhanced Services data group, which was the authorised “guardian” of this data set, granted the permission to analyse the data.

### Study design and setting

The data reported in this paper comes from the West of Scotland, with a population of circa 1.8 million served by two different health boards. The local health boards oversee a programme of incentivised depression screening in chronic disease as part of a wider chronic disease management programme of ‘Local Enhanced Services’ (LES). These are contractual arrangements at a local health board level with family practices where incentivisation is offered to primary care practitioners on certain indicators of chronic disease management. However, there are no penalties for non-adherence. In the areas under investigation in our study, family practices were paid under the LES scheme to carry out a comprehensive annual health assessment, which included depression screening, for patients with three common cardiometabolic conditions, CHD, diabetes and stroke. The annual health assessment was usually carried out by a practice nurse and lasted approximately one hour. The protocol for health assessment was specific for each of the three diseases but included monitoring of blood pressure (BP), total cholesterol, body mass index (BMI) and in those with diabetes, HbA1c. The assessment included detailed history taking, various physical examinations and blood tests.

### Participants

We restricted our analysis to adults aged from 18 to 90 and health assessments recorded between 01/04/2008 to 31/03/2009. A total of 125,143 patients were listed as having CHD, diabetes or stroke in the year 2008–09, the “DepChron” dataset [14], described in a previous publication. Of the total sample, 10,670 (8.5%) patients were under treatment for depression and were thus exempt from screening. The remaining 114,473 (91.5% of total sample size) patients were eligible for depression screening. However, the uptake of depression screening was poor and only undertaken in 35,537 (31.1% of those eligible) and 78,936 (68.9%) were not screened.

### Measurement of clinical risk factors and outcome variable

Systolic blood pressure (SBP) and diastolic blood pressure (DBP) measurements were recorded in mm Hg and BMI in kg/m^2^ determined from height and weight measurements. A blood sample was collected by the practice nurse at the time of assessment; the result for total cholesterol was reported in mmol/l and HbA1c was reported in Diabetes Control and Complications Trial (DCCT) units.

We restricted the values for cardiovascular risk factors to a clinically plausible range based on both our clinical judgement and the findings of general population studies. SBP measurements were restricted to a range between 90 to 240 mm Hg and DBP to a range between 50 to 130 mm Hg [22, 23]. Similarly, BMI was restricted to a range between 15 to55 [24], total cholesterol to 2–10 [25] and HbA1C to 3-18% [26]. Observations in the data which were outside these range were excluded from the analysis. The depression subscale of HADS (HADS-D) gives a total score of 0 to 21, and a threshold of >7 was used to define the presence of depressive symptoms, as endorsed by national guidelines [27]. The area based Scottish Index of Multiple Deprivations (SIMD) was used as a measure of socioeconomic status [28].

### Statistical analysis

We used multiple logistic regression with the outcome variable as the prevalence of a positive screening for depression (defined as HADS-D >7). We used five separate regression models to examine the impact of each individual cardiovascular risk factor (SBP, DBP, total cholesterol, BMI, HbA1c) on the odds of a raised HADS-D. We entered quadratic terms for each clinical measure into regression models to allow for a non-linear relationship. We entered age (18–64 vs. 65–90), sex (male vs. female) and socio-economic status (deprived: SIMD deciles 1–5 vs. affluent: SIMD deciles 6–10) into all of the models as binary variables. We also included the number of comorbid conditions (range 1–3, representing a combination of one or more of the three cardiometabolic disease under investigations: CHD, stroke or diabetes) into all regression models as a categorical variable. We present the results as a graph of the predicted probability of a raised HADS-D against corresponding values of the clinical risk factor. We calculated the turning point for each risk factor using the formula min = -b/2a where “a” represents coefficient of quadratic term and “b” represents coefficient of linear term.

We used the R statistical software, version 3.0.2 for statistical analysis [29].

### Supplementary and sensitivity analyses

The screened population was a subset of the whole dataset and the majority of the patients eligible for depression screening did not have HADS-D recorded due to poor uptake of depression screening. We compared the demographic features and distribution of clinical risk factors in both the screened population and the total population. We tested for interactions of each clinical risk factor with age, gender, number of comorbid condition and deprivation status for each of the corresponding regression models to check for potential effect modification. We also tested for cubic terms in each of the five regression models for five clinical measures. Sensitivity of the results to excluded values was assessed by repeating the analyses with all available patients.

We also performed multiple linear regression analysis with HADS-D as a continuous scale. We used five separate regression models to examine the impact of each individual cardiovascular risk factor (SBP, DBP, total cholesterol, BMI, HbA1c) on HADS-D as a continuous scale after excluding extreme values for each clinical measure as defined above. Quadratic and cubic terms for each clinical measure and other predictor variables, such as age, sex, socio-economic status and number of co-morbid conditions were added to the linear regression model as described above. The turning point or the “nadir” was calculated using the same formula described in the preceding section.

## Results

### Sample size and characteristics

**Figure 1:**
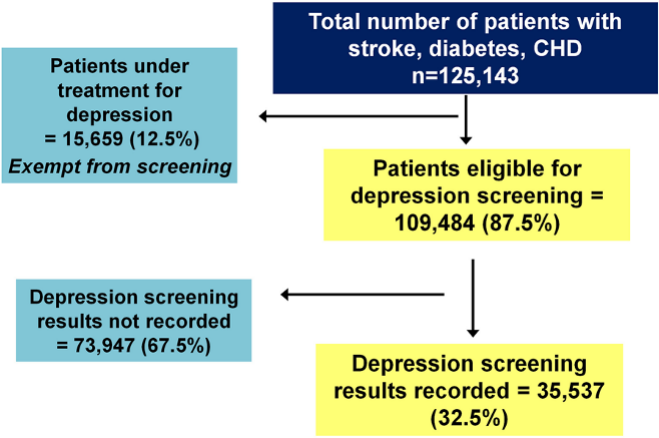
**Study sample size and recruitment.**

N = 35,537 (32.5% of total population) patients with one of the three chronic cardiometabolic diseases CHD, previous stroke and diabetes had results of depression screening with HADS-D recorded (see Figure 1). The demographic characteristics and cardiovascular risk factor distribution between the screened and total population were similar (please see “Additional file 1-Additional Analysis”). The HADS-D was positive (>7) for 7080 patients (19.9%). The demographic characteristics of the study sample are described in Table 1.

**Table 1:** **Patient demographics of the study sample**

	Demographics	DepChron (n=35,537)
Age group	***Missing***	11
	**18-64**	11553 (32.52%)
	**64-90**	23973 (67.48%)
Gender	***Missing***	18
	**Male**	20658 (58.16%)
	**Female**	14861 (41.84%)
Deprivation status	***Missing***	732
	**Deprived**	22726 (65.30%)
	**Affluent**	12079 (34.70%)
Number of comorbid condition	***Missing***	0
	**One**	27356 (76.99%)
	**Two**	7410 (20.85%)
	**Three**	771 (2.16%)

The distributions of the five cardiovascular risk factors in the study sample such as sample mean, standard deviation, missing values and the observations outside the plausible range considered and observed nadirs are described in Table 2.

**Table 2:** **Study sample (n = 35537) distribution for the five clinical risk factors, missing values, the number of extreme observations outside the usually observed clinically plausible values, analyzed data and observed nadirs**

Clinical measure (range included)	Mean (SD)	N missing	Exclusions	N analyzed	Observed nadirs
Systolic BP (90–240)	133 (17.54)	3398	n <90 = 110	32029	148 mm Hg
			n >240 = 0		
Diastolic BP (50–130)	74 · 57 (10.32)	3398	n <50 = 165	31972	74 mm Hg
			n >130 = 2		
Body mass index (15–55)	28 · 95 (6.02)	5398	n <15 = 29	30042	30.70 kg/m2
			n >55 = 68		
Total cholesterol (2–10)	4 · 31 (1.05)	4226	n <2 = 50	31244	3.60 mmol/l
			n >10 = 17		
HbA1c (3–18)	7 · 52 (1.68)	2775	n <3 = 2	15676	7.06 DCCT
			n >18 = 0		

Legend: BP = Blood Pressure; n = 18453 for HbA1c.

### Blood pressure, total cholesterol, body mass index and depression

**Figure 2:**
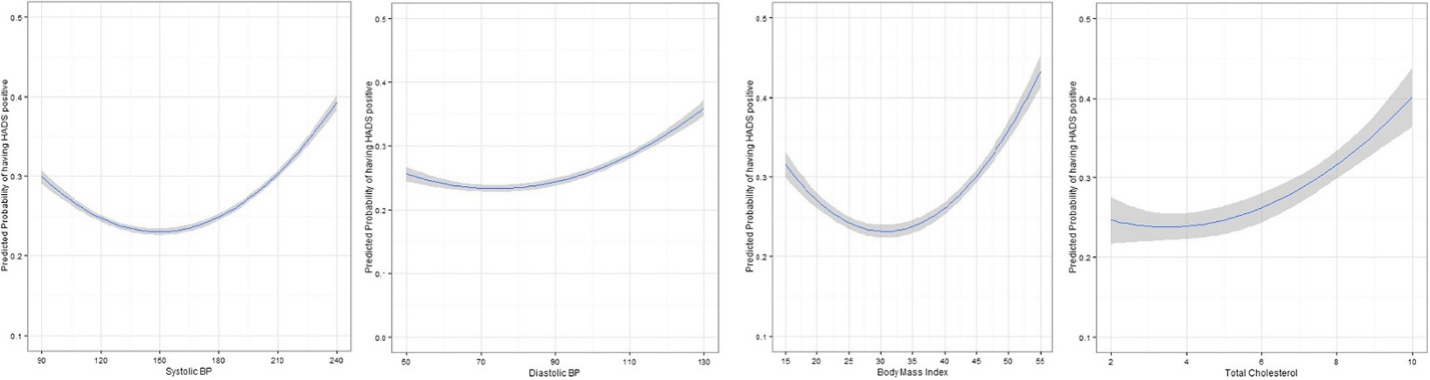
**Relationship of Systolic BP (SBP), Diastolic BP (DBP), Body Mass Index (BMI) and total cholesterol with probability of having a positive HADS-D (>7) with 95% confidence intervals.**

SBP was found to have a “J-shaped” relationship with the probability of having a positive result with HADS-D screening, based on a regression model using all of the screened population with at least one of the three chronic diseases. The nadir or the minimum level of SBP with the least probability of having a positive screening result with HADS-D was found to be 148 mm Hg (see Figure 2). DBP was found to have a “J-shaped” relationship with the probability of having a positive result with HADS-D screening, based on a regression model using all of the screened population with at least one of the three chronic diseases. However, the shape of the J-curve was shallow for DBP when compared with SBP. The nadir for DBP with the least probability of having a positive screening result with HADS-D was found to be 74 mm Hg. This observed relationship between SBP, DBP and depressive symptoms remained significant after adjusting for age, sex, number of comorbid conditions and socio-economic status.

BMI was found to have a non-linear relationship with the probability of having a positive result with HADS-D screening, based on a regression model using all of the screened population with at least one of the three cardiometabolic conditions. The nadir or the minimum level for BMI was found to be 30.70 kg/m^2^ (see Figure 2). Total Cholesterol was found to have a non-linear relationship with the probability of having a positive result with HADS-D screening (HADS-D > 7), based on the same regression model described above. However, the shape of the curve was less pronounced with lower values of total cholesterol and wider confidence intervals when compared to SBP and BMI. The nadir or the minimum level for total cholesterol was found to be 3.60 mmol/l (see Figure 2). The relationship between BMI and total cholesterol with probability of having HADS-D positive remained significant after adjusting for age, sex, number of comorbid conditions and socio-economic status.

### HbA1C and depression

**Figure 3:**
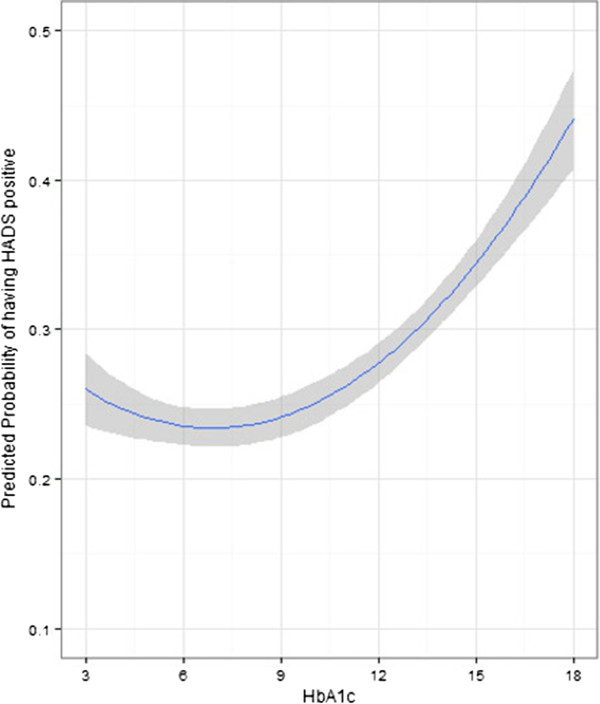
**Relationship of HbA1C with probability of having a positive HADS-D (>7) with 95% confidence intervals.**

HbA1c was found to have a non-linear “J-shaped” relationship with the probability of having a positive result with HADS-D screening (HADS-D > 7), based on a regression model using only patients with diabetes (n = 18,453, missing = 2775, excluded = 2). The shape of the curve was more similar but the confidence intervals were slightly wider, when compared to SBP, DBP and BMI. The nadir or the minimum level for HbA1c was found to 7.06% DCCT (54 mmol/mol IFCC) (see Figure 3). This relationship also remained significant after adjusting for age, sex, number of comorbid conditions and socio-economic status.

### Supplementary and sensitivity analysis

There were no significant cubic terms for any of the cardiovascular risk factors. There were significant interactions between DBP and sex (p-value = 0.01) and BMI and age (p-value = 0.009) (please see “Additional file 1-Additional Analysis”). Hence, we calculated the nadirs separately for these groups with significant interactions. The nadirs for DBP were 78 mm Hg for males and 63 mm Hg for females respectively. The nadirs for BMI were 32.12 kg/m2 for those aged18-64 years and 29.54 kg/m2 for those 65–90 years respectively. The shape of the curve was unchanged for DBP and BMI after doing sub-group analysis for sex and age respectively (please see “Additional file 1-Additional Analysis”). The results were unchanged after including extremes of clinical values outside the clinically plausible range described above (please see “Additional file 1-Additional Analysis”).

The five cardiovascular risk factors had a non-linear relationship in the respective linear regression models, after adjusting for age, sex, socio-economic status and number of co-morbid conditions. The observed nadirs for SBP, DBP, total cholesterol and BMI were 145 mm Hg, 78 mm Hg, 3.41 mmol/l and 30.25 kg/m2 respectively. The observed nadir for HbA1c in patients with diabetes was 6.21 DCCT (44.4 mmol/mol IFCC). The value for HADS-D increased with increase in value of these clinical measures above their respective nadirs but it increased with decrease in value below these observed nadirs. There were no significant cubic terms. The results of each linear regression are presented in detail in “Additional file 2- Linear Regression with HADS-D as continuous measure”.

## Discussion

In a large, community based sample of patients with CHD, previous stroke, or diabetes depressive symptoms assessed using depression screening were found to have a nonlinear association with five routine cardiovascular risk factors of disease management. The relationships were ‘J-shaped’ with high levels of SBP and BMI associated with greater levels of concurrent depressive symptoms, but with the lowest levels also associated with increased prevalence of depressive symptoms. DBP and total Cholesterol had a similar but weaker relationship with depression. In patients with diabetes, a “J-shaped” relationship was again observed between HbA1c levels and depressive symptoms. These associations remained significant after adjusting for demographic factors such as age, sex, number of comorbid conditions and socio-economic status; including or excluding clinical observations with extreme values and using HADS-D as continuous scale.

Previous evidence studying the relationship between cardiovascular risk factor values and depressive symptoms has mainly come from general population studies. Barrett-Connor et al. reported a non-linear relationship between DBP and depression with a observed nadir of 75 mm Hg DBP for concurrent depressive symptoms in a general population sample [30]. In various cross-sectional studies involving mainly elderly population, depression has been observed to have a non-linear association with SBP [31–34] and DBP [30, 35]. Similarly, increased prevalence of depressive symptoms has been observed with extreme values of total cholesterol [36, 37] and HbA1c in general population samples [38], in a non-linear trend. There is as yet no published literature that we know of that examines the relationship between cardiovascular risk factors and depressive symptoms in those with cardiometabolic disease.

Non-linear relationship between extreme values of SBP, DBP, BMI and HbA1c and adverse clinical outcomes such as increased incidence of vascular events and deaths in patients with cardiometabolic conditions has been reported extensively [39–43].

There are two potential implications of our findings. Firstly, if the association between extreme values of these risk factors with depressive symptoms in those with cardiometabolic disease is supported by prospective studies, then this relationship could be used to identify those at “high risk” of depression. This would then offer a mechanism for targeting of depression screening in those with cardiometabolic disease. Secondly, these results need to be replicated using other datasets and also prospectively to further explain the nature and direction of the observed association between depressive symptoms and cardiovascular risk factors values. Such further investigation is necessary in order to determine whether the lower cardiovascular risk factors are merely markers of other disease processes (for example, low total cholesterol levels associated with malnutrition, liver diseases and haematological diseases) [44–46] that may make patients more vulnerable to experiencing depressive symptoms or whether it could be attributed to a potential side-effect of aggressive cardiovascular risk factor management [47–50].

This study has a number of key strengths. The data came from a large, community based sample, and importantly reflecting real life clinical practice. There are several limitations. As the study was based on cross-sectional analysis, it is not possible to make causal inferences from the findings of this study. It is therefore unclear whether the observed non-linear association of cardiovascular risk factors with prevalent depressive symptoms is due to cause or effect.

Secondly, we did not have complete information on biobehavioural factors such as smoking status, alcohol intake and levels of physical activity which are likely to influence the values of cardiovascular risk factors considered and also the prevalence of depressive symptoms [51–54].

Since only a minority of the patients were actually screened, depression status was unknown for a large number of patients, which remains an important limitation. There may be important differences between patients with known depression status and those whose depression status was unknown, which are not clearly evident from their baseline demographic data. Practitioners may intuitively screen those patients where they are more likely to get a positive result, for instance patients with multimorbidity. Also, there is a possibility of reverse causality with GPs reviewing a patient whom they consider to have depression and offering screening subsequently. Previously reported barriers to discussing depression (or mental health) in patients with chronic disease in primary care, such as stigma associated around the ‘label’ and physicians’ preconception of normalizing depression in patients with chronic disease, could be influencing factors behind low uptake of depression screening in our study [55, 56].

Finally, the overall accuracy of depression screening in our study was reliant on HADS-D which is a self-reported measure and it is not a gold standard measure for assessing depressive symptoms in patients with cardiometabolic disease in a primary care setting [11, 57, 58]. We also did not have information on history of previous episodes of depression for patients in our study which may influence the prevalence levels for depressive symptoms.

## Conclusion

In a general practice sample of patients with CHD, stroke, or diabetes, depressive symptoms were found to have a strong curvilinear association with SBP, BMI, and HbA1c; and a weaker curvilinear association with total cholesterol and DBP. Further investigation of these relationships is urgently needed to clarify the nature of these associations, in order to determine whether they have potentially important implications for clinical practice in relation to either risk stratification for depression or our approach to secondary prevention in individuals with cardiometabolic disease.

## Electronic supplementary material

Additional file 1: **“Additional Analysis”.** Results of sensitivity and supplementary analysi. (PDF 215 KB)

Additional file 2: **“Linear Regression with HADS-D as continuous measure”.** Results of statistical analysis with linear regression models using HADS-D as a continuous measure. (PDF 183 KB)
