# Supplementary material for: Revisiting the J shaped curve, exploring the association between cardiovascular risk factors and concurrent depressive symptoms in patients with cardiometabolic disease: Findings from a large cross-sectional study
Source: BMC Cardiovasc Disord. 2014 Oct 28;14:139. doi: 10.1186/1471-2261-14-139 (PMC4271339; doi:10.1186/1471-2261-14-139)
Supplement: Supplementary file 1 — Additional file 1: “Additional Analysis”. Results of sensitivity and supplementary analysi. (PDF 215 KB) [file 12872_2014_820_MOESM1_ESM.pdf]

Revisiting the J shaped curve, exploring the association between cardiovascular risk factors and concurrent depressive symptoms in patients with cardiometabolic disease: Findings from a large cross-sectional study.

Supplement 1 - Additional Analysis

Table 1 Title: Comparison of demographic characteristics and distribution of clinical risk factors between total population (n=125143) with the subset which underwent depression screening (n=35537)

Mean (SD) and median (IQR) are presented for continuous variables and count (%) for categorical.			
		Total Population n=125143	Screened Population n=35537
**Age (years)**	18-64<br>65-90<br>missing	47711 (38.13%)<br>77390 (62.87%)<br>42	11553 (32.52%)<br>23973 (67.48%)<br>11
**Sex**	Female<br>Male<br>missing	57566 (46.03%)<br>67507 (53.97%<br>70	14861 (41.84%)<br>20658 (58.16%)<br>18
**Socio-economic status**	Deprived<br>Affluent<br>missing	82267 (67.46%)<br>39680 (33.54%)<br>3196	22726 (65.30%)<br>12079 (34.70%)<br>732
**Comorbid Condition**	One<br>Two<br>Three<br>missing	101219 (80.88%)<br>21666 (17.32%)<br>2258 (1.80%)<br>0	27356 (76.99%)<br>7410 (20.85%)<br>771 (2.16%)<br>0
**Systolic Blood Pressure**	Mean (SD)<br>Median (IQR)<br>missing	133.9 (18.30)<br>133 (23)	133 (17.54)<br>132 (22)
**Diastolic Blood Pressure**	Mean (SD)<br>Median (IQR)<br>missing	75.57 (10.74)<br>76 (11)	74.57 (10.32)<br>75 (11)
**Body Mass Index**	Mean (SD)<br>Median (IQR)<br>missing	29.06 (6.22)<br>28.26 (7.37)	28.95 (6.02)<br>28.09 (7.10)
**Total Cholesterol**	Mean (SD)<br>Median (IQR)<br>missing	4.43 (1.12)<br>4.30 (1.3)	4.31 (1.05)<br>4.2 (1.2)
**HbA1c**	Mean (SD)<br>Median (IQR)<br>missing	7.72 (1.82)<br>7.30 (2.2)	7.52 (1.68)<br>7.1 (1.8)

Systolic Blood Pressure (SBP)-Additional Analysis

Table 2: Title: Comparison of results of multiple logistic regression for outcome of concurrent depressive symptoms (HADS-D>7) with and without extreme values for Systolic Blood Pressure. Legend: SBP: Systolic Blood Pressure. Regression 1a shows the regression of analyzed data after excluding extreme values for SBP (SBP <90 mm Hg and SBP >240 mm Hg). Regression 1b shows results of data including extreme values.

	**Regression 1a- Analyzed Data without extreme values N=32029**		**Regression 1b- All available data including extreme values N=32139**	
**Variable**	Odds Ratio with 95% Confidence Interval	p-value	Odds Ration with 95% Confidence Interval	p-value
**SBP**	0.96 (0.94-0.97)	<0.001	0.96 (0.94-0.97)	<0.001
**SBP ^2**	1.00013 (1.00008-1.00018)	<0.001	1.00013 (1.00008-1.00018)	<0.001
**Age Group(65-90)**	0.57 (0.53-0.60)	<0.001	0.57 (0.53-0.60)	<0.001
**Sex (Male)**	0.82 (0.77-0.87)	<0.001	0.82 (0.77-0.87)	<0.001
**Deprivation Status (Affluent)**	0.50 (0.46-0.53)	<0.001	0.50 (0.46-0.53)	<0.001
**Co-morbid Conditions**				
**Two**	1.45 (1.36-1.56)	<0.001	1.45 (1.36-1.55)	<0.001
**Three**	1.65 (1.37-1.94)	<0.001	1.65 (1.38-1.96)	<0.001

Table 3: Title: Statistical significance of interaction between Systolic Blood Pressure and various demographic factors in multiple logistic regression for outcome of concurrent depressive symptoms (HADS-D >7). Legend: SBP: Systolic Blood Pressure

	**Regression 1a- Analyzed Data without extreme values N=32029**
**Interaction**	p-value
**SBP: Age Group**	0.42
**SBP: Deprivation Status**	0.06
**SBP: Sex**	0.12
**SBP: Co-morbid Condition**	
**Two**	0.67
**Three**	0.70

Diastolic Blood Pressure (DBP)-Additional Analysis

Table 4: Title: Comparison of results of multiple logistic regression for outcome of concurrent depressive symptoms (HADS-D>7) with and without extreme values for Diastolic Blood Pressure. Legend: DBP: Diastolic Blood Pressure. Regression 2a shows the regression of analyzed data after excluding extreme values for DBP (SBP <50 mm Hg and SBP >130 mm Hg). Regression 2b shows results of data including extreme values.

	**Regression 2a- Analyzed Data without extreme values N=31972**		**Regression 2b- All available data including extreme values N=32139**	
**Variable**	Odds Ratio with 95% Confidence Interval	p-value	Odds Ration with 95% Confidence Interval	p-value
**DBP**	0.95 (0.93-0.98)	0.001	0.96 (0.94-0.99)	0.005
**DBP ^2**	1.0002 (1.0001-1.0004)	0.001	1.0002 (1.00007-1.0003)	0.003
**Age Group(65-90)**	0.56 (0.53-0.60)	<0.001	0.56 (0.53-0.60)	<0.001
**Sex (Male)**	0.82 (0.77-0.87)	<0.001	0.82 (0.77-0.87)	<0.001
**Deprivation Status (Affluent)**	0.49 (0.46-0.53)	<0.001	0.50 (0.46-0.53)	<0.001
**Co-morbid Conditions**				
**Two**	1.46 (1.36-1.56)	<0.001	1.47 (1.37-1.57)	<0.001
**Three**	1.64 (1.38-1.95)	<0.001	1.64 (1.37-1.94)	<0.001

Table 5: Title: Statistical significance of interaction between Diastolic Blood Pressure and various demographic factors in multiple logistic regression for outcome of concurrent depressive symptoms (HADS-D >7). Legend: DBP: Diastolic Blood Pressure

	**Regression 2a- Analyzed Data without extreme values N=31972**
**Interaction Term**	p-value
**DBP: Age Group**	0.23
**DBP: Deprivation Status**	0.85
**DBP: Sex**	**0.01 (Significant)**
**DBP: Co-morbid Condition**	
**Two**	0.55
**Three**	0.93

Figure 1: Title: Relationship between Diastolic Blood Pressure (DBP) and probability of having HADS-D positive with 95% confidence intervals with sub-group analysis for males and females.

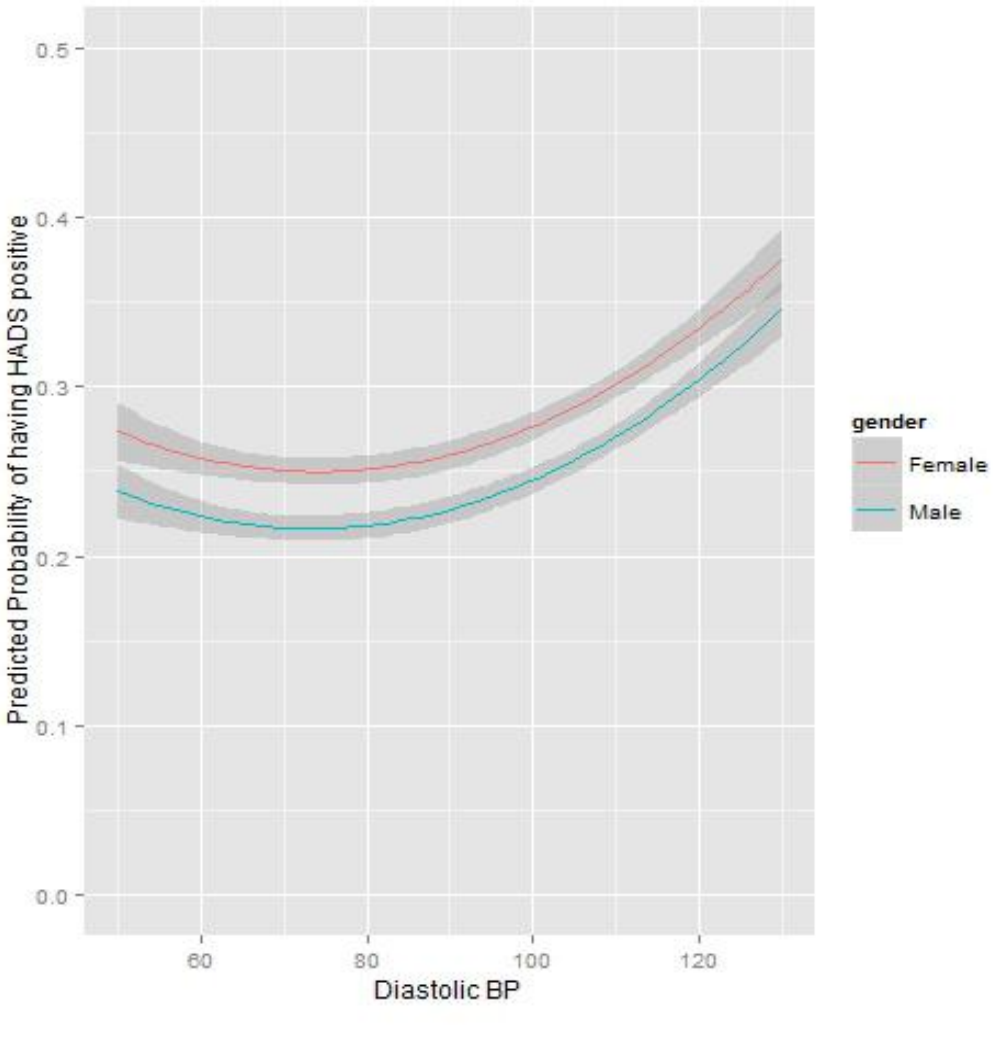

<u>Total Cholesterol-Additional Analysis</u>

Table 6: Title: Comparison of results of multiple logistic regression for outcome of concurrent depressive symptoms (HADS-D>7) with and without extreme values for Total Cholesterol. Legend: Regression 3a shows the regression of analyzed data after excluding extreme values for Total Cholesterol (Total Cholesterol < 2 mmol/l and Total Cholesterol > 10 mmol/l). Regression 3b shows results of data including extreme values.

	**Regression 3a- Analyzed Data without extreme values N=31244**		**Regression 3b- All available data including extreme values N=31311**	
**Variable**	Odds Ratio with 95% Confidence Interval	p-value	Odds Ration with 95% Confidence Interval	p-value
**Cholesterol**	0.79 (0.69-0.92)	0.008	0.88 (0.78-1.00)	0.06
**Cholesterol ^2**	1.03 (1.01-1.04)	0.002	1.019 (1.007-1.032)	0.003
**Age Group(65-90)**	0.57 (0.54-0.61)	<0.001	0.57 (0.54-0.61)	<0.001
**Sex (Male)**	0.85 (0.80-0.90)	<0.001	0.85 (0.80-0.90)	<0.001
**Deprivation Status (Affluent)**	0.50 (0.46-0.53)	<0.001	0.50 (0.46-0.53)	<0.001
**Co-morbid Conditions**				
**Two**	1.50 (1.40-1.60)	<0.001	1.50 (1.40-1.60)	<0.001
**Three**	1.74 (1.45-2.07)	<0.001	1.75 (1.46-2.09)	<0.001

Table 7: Title: Statistical significance of interaction between Total Cholesterol and various demographic factors in multiple logistic regression for outcome of concurrent depressive symptoms (HADS-D >7)

	**Regression 3a- Analyzed Data without extreme values N=31244**
**Interaction Term**	p-value
**Cholesterol: Age Group**	0.22
**Cholesterol: Deprivation Status**	0.17
**Cholesterol: Sex**	0.65
**Cholesterol: Co-morbid Condition**	
**Two**	0.63
**Three**	0.95

Body Mass Index-Additional Analysis

Table 8: Title: Comparison of results of multiple logistic regression for outcome of concurrent depressive symptoms (HADS-D>7) with and without extreme values for Body Mass Index. Legend: BMI: Body Mass Index. Regression 4a shows the regression of analyzed data after excluding extreme values for BMI (BMI < 15 kg/m2 and BMI > 55 kg/m2). Regression 4b shows results of data including extreme values.

	**Regression 4a- Analyzed Data without extreme values N=30042**		**Regression 4b- All available data including extreme values N=30139**	
**Variable**	Odds Ratio with 95% Confidence Interval	p-value	Odds Ration with 95% Confidence Interval	p-value
**BMI**	0.85 (0.82-0.88)	< 0.001	0.96 (0.94-0.98)	0.009
**BMI ^2**	1.002 (1.001-1.003)	< 0.001	1.0006 (1.00003-1.0009)	< 0.001
**Age Group(65-90)**	0.57 (0.53-0.61)	<0.001	0.56 (0.52-0.60)	<0.001
**Sex (Male)**	0.87 (0.82-0.92)	<0.001	0.85 (0.80-0.90)	<0.001
**Deprivation Status (Affluent)**	0.52 (0.48-0.55)	<0.001	0.52 (0.48-0.55)	<0.001
**Co-morbid Conditions**				
**Two**	1.50 (1.40-1.60)	<0.001	1.49 (1.39-1.60)	<0.001
**Three**	1.80 (1.51-2.13)	<0.001	1.78 (1.49-2.11)	<0.001

Table 9: Title: Statistical significance of interaction between Body Mass Index and various demographic factors in multiple logistic regression for outcome of concurrent depressive symptoms (HADS-D >7). Legend: BMI: Body Mass Index

	**Regression 4a- Analyzed Data without extreme values N=30042**
**Interaction Term**	p-value
**BMI: Age Group**	**0.009 (Significant)**
**BMI: Deprivation Status**	0.13
**BMI: Sex**	0.07
**BMI: Co-morbid Condition**	
**Two**	0.06
**Three**	0.40

Figure 2: Title: Relationship between Body Mass Index (BMI) and probability of having HADS-D positive with 95% confidence intervals with sub-group analysis for the two age-groups.

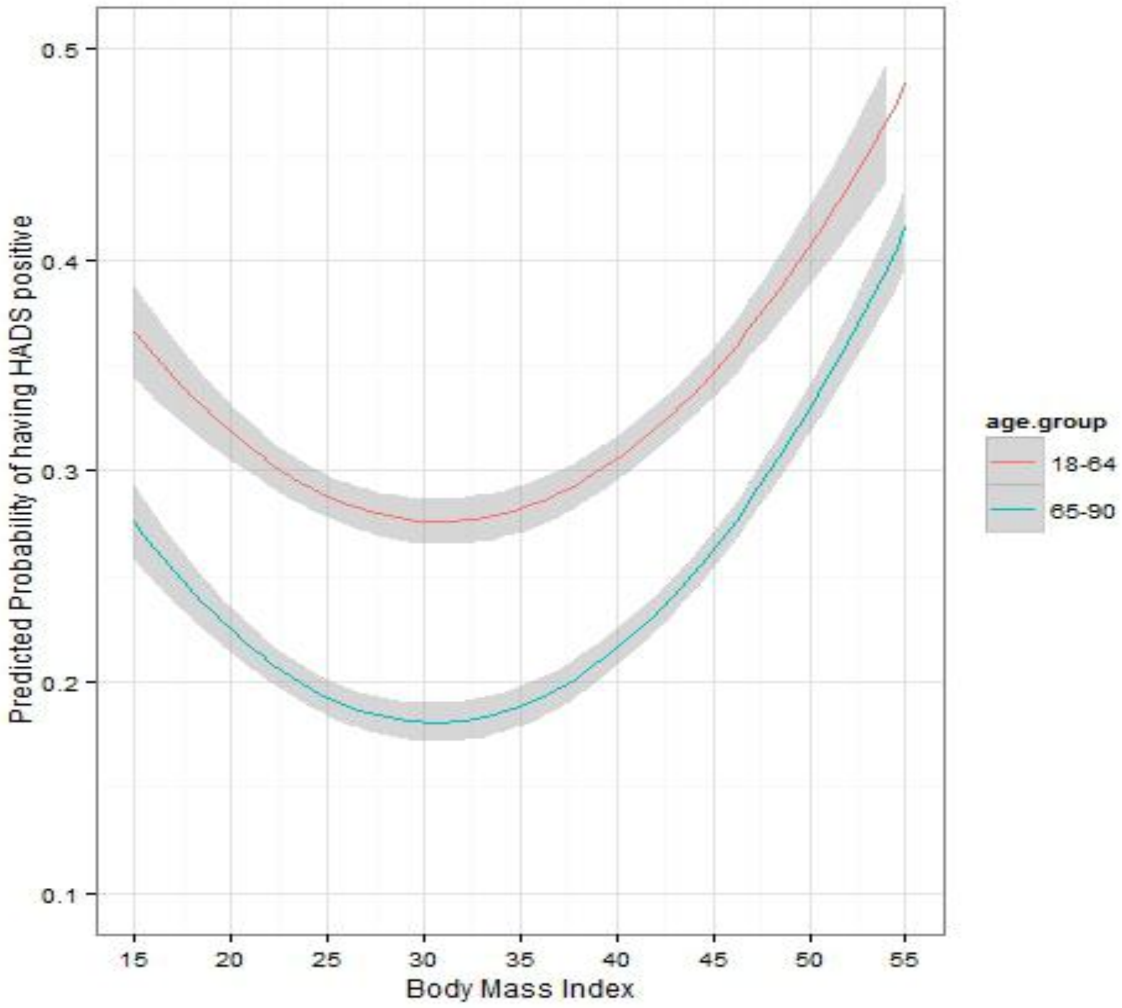

HbA1c-Additional Analysis

Table 10: Title: Comparison of results of multiple logistic regression for outcome of concurrent depressive symptoms (HADS-D>7) with and without extreme values for HbA1c. Legend: Regression 5a shows the regression of analyzed data after excluding extreme values for HbA1c (HbA1c < 3 DCCT and HbA1c>18 DCCT). Regression 5b shows results of data including extreme values.

	**Regression 5a- Analyzed Data without extreme values N=15676**		**Regression 5b- All available data including extreme values N=15678**	
**Variable**	Odds Ratio with 95% Confidence Interval	p-value	Odds Ratio with 95% Confidence Interval	p-value
**HbA1c**	0.82 (0.70-0.95)	0.01	0.82 (0.71-0.96)	0.01
**HbA1c^2**	1.014 (1.005-1.022)	0.001	1.014 (1.005-1.022)	0.001
**Age Group(65-90)**	0.58 (0.53-0.63)	<0.001	0.58 (0.53-0.63)	<0.001
**Sex (Male)**	0.82 (0.76-0.89)	<0.001	0.82 (0.76-0.89)	<0.001
**Deprivation Status (Affluent)**	0.52 (0.48-0.58)	<0.001	0.52 (0.48-0.58)	<0.001
**Co-morbid Conditions**				
**Two**	1.68 (1.40-1.60)	<0.001	1.68 (1.40-1.60)	<0.001
**Three**	1.99 (1.51-2.13)	<0.001	1.99 (1.51-2.13)	<0.001

Table 11: Title: Statistical significance of interaction between Body Mass Index and various demographic factors in multiple logistic regression for outcome of concurrent depressive symptoms (HADS-D >7).

	**Regression 5a- Analyzed Data without extreme values N=15676**
**Interaction Term**	p-value
**HbA1c: Age Group**	0.28
**HbA1c: Deprivation Status**	0.22
**HbA1c: Sex**	0.08
**HbA1c: Co-morbid Condition**	
**Two**	0.45
**Three**	0.92
