# Supplementary material for: Revisiting the J shaped curve, exploring the association between cardiovascular risk factors and concurrent depressive symptoms in patients with cardiometabolic disease: Findings from a large cross-sectional study
Source: BMC Cardiovasc Disord. 2014 Oct 28;14:139. doi: 10.1186/1471-2261-14-139 (PMC4271339; doi:10.1186/1471-2261-14-139)
Supplement: Supplementary file 2 — Additional file 2: “Linear Regression with HADS-D as continuous measure”. Results of statistical analysis with linear regression models using HADS-D as a continuous measure. (PDF 183 KB) [file 12872_2014_820_MOESM2_ESM.pdf]

Revisiting the J shaped curve, exploring the association between cardiovascular risk factors and concurrent depressive symptoms in patients with cardiometabolic disease: Findings from a large cross-sectional study.

Supplement 2 – Linear Regression with HADS-D as Continuous Measure

Systolic Blood Pressure (SBP)- Linear Regression Analysis

Table 1: Title: Results of multiple linear regression for outcome of concurrent depressive symptoms (HADS-D) without extreme values for Systolic Blood Pressure. Legend: SBP: Systolic Blood Pressure. The table shows regression of analysed data after excluding extreme values for SBP (SBP <90 mm Hg and SBP >240 mm Hg) with HADS-D as a continuous variable. HADS-D was transformed into square root (HADS-D) as it was not normally distributed.

	**Regression 1- Analysed Data without extreme values N=32029**		
**Variable**	Regression co-efficient	p-value	Standard Error
**SBP**	-1.61	<0.001	2.94
**SBP ^2**	5.57	<0.001	1.06
**Age Group(65-90)**	-9.06	<0.001	1.16
**Sex (Male)**	-1.36	<0.001	1.08
**Deprivation Status (Affluent)**	-2.82	<0.001	1.11
**Co-morbid Conditions**			
**Two**	1.86	<0.001	1.29
**Three**	3.05	<0.001	3.55

Diastolic Blood Pressure (DBP)-Linear Regression Analysis

Table 2: Title: Results of multiple linear regression for outcome of concurrent depressive symptoms (HADS-D) without extreme values for Diastolic Blood Pressure. Legend: DBP: Diastolic Blood Pressure. The table shows the regression of analysed data after excluding extreme values for DBP (DBP <50 mm Hg and DBP >130 mm Hg) with HADS-D as a continuous variable. HADS-D was transformed into square root (HADS-D) as it was not normally distributed.

	**Regression 2- Analysed Data without extreme values N=31972**		
**Variable**	Regression co-efficient	p-value	Standard Error
**DBP**	-1.90	<0.001	5.10
**DBP ^2**	1.21	<0.001	3.33
**Age Group(65-90)**	-9.66	<0.001	1.19
**Sex (Male)**	-1.35	<0.001	1.08
**Deprivation Status (Affluent)**	-2.83	<0.001	1.11
**Co-morbid Conditions**			
**Two**	1.86	<0.001	1.29
**Three**	3.03	<0.001	3.57

Total Cholesterol-Linear Regression Analysis

Table 3: Title: Results of multiple linear regression for outcome of concurrent depressive symptoms (HADS-D) without extreme values for Total Cholesterol. Legend: The table shows the regression of analysed data after excluding extreme values for Total Cholesterol (Total Cholesterol < 2 mmol/l and Total Cholesterol > 10 mmol/l) with HADS-D as a continuous variable. HADS-D was transformed into square root (HADS-D) as it was not normally distributed. Total Cholesterol was log transformed as it was not normally distributed.

	**Regression 3- Analysed Data without extreme values N=31244**		
**Variable**	Regression co-efficient	p-value	Standard Error
**Log (total cholesterol)**	-0.90	<0.001	0.19
**Log (total cholesterol) ^2**	0.35	<0.001	0.06
**Age Group(65-90)**	-0.07	<0.001	0.01
**Sex (Male)**	-0.12	<0.001	0.01
**Deprivation Status (Affluent)**	-0.28	<0.001	0.01
**Co-morbid Conditions**			
**Two**	0.19	<0.001	0.01
**Three**	0.32	<0.001	0.03

Body Mass Index-Linear Regression Analysis

Table 4: Title: Results of multiple linear regression for outcome of concurrent depressive symptoms (HADS-D) without extreme values for Body Mass Index. Legend: BMI: Body Mass Index. The table shows the regression of analysed data after excluding extreme values for BMI (BMI < 15 kg/m2 and BMI > 55 kg/m2) with HADS-D as a continuous variable. HADS-D was transformed into square root (HADS-D) as it was not normally distributed. BMI was log transformed as it was not normally distributed.

	**Regression 4- Analysed Data without extreme values N=30042**		
**Variable**	Regression co-efficient	p-value	Standard Error
**Log (BMI)**	-9.51	<0.001	0.67
**Log (BMI) ^2**	1.41	<0.001	0.10
**Age Group(65-90)**	-0.08	<0.001	0.01
**Sex (Male)**	-0.11	<0.001	0.01
**Deprivation Status (Affluent)**	-0.27	<0.001	0.01
**Co-morbid Conditions**			
**Two**	0.18	<0.001	0.01
**Three**	0.33	<0.001	0.03

HbA1c-Linear Regression Analysis

Table 5: Title: Results of multiple linear regression for outcome of concurrent depressive symptoms (HADS-D) without extreme values for HbA1c. Legend: The table shows the regression of analysed data after excluding extreme values HbA1c (HbA1c < 3 DCCT and HbA1c>18 DCCT). Regression 5b shows results of data including extreme values with HADS-D as a continuous variable. HADS-D was transformed into square root (HADS-D) as it was not normally distributed. HbA1c was log transformed as it was not normally distributed.

	**Regression 5- Analysed Data without extreme values N=15676**		
**Variable**	Regression co-efficient	p-value	Standard Error
**Log (HbA1c)**	-2.13	<0.001	0.55
**Log (HbA1c) ^2**	0.55	<0.001	0.13
**Age Group(65-90)**	-0.07	<0.001	0.01
**Sex (Male)**	-0.12	<0.001	0.01
**Deprivation Status (Affluent)**	-0.25	<0.001	0.01
**Co-morbid Conditions**			
**Two**	0.24	<0.001	0.01
**Three**	0.40	<0.001	0.03
